# Development and validation of the Illness Representation Interview (IRI)

**DOI:** 10.1192/j.eurpsy.2024.242

**Published:** 2024-08-27

**Authors:** D. Y. Kim, H. Y. Shin, S. Choi

**Affiliations:** Clinical Psychology, Duksung Women’s University, Seoul, Korea, Republic Of

## Abstract

**Introduction:**

It has been several years since the World Health Organization (WHO) advocated for shared decision-making(SDM) models when developing treatment plans for individuals with mental illnesses. It is emphasizing the importance of actively involving patients in expressing their opinions and sharing treatment-related information. However, few clinicians accept patients’ subjective views in clinical practice. Given that patients’ subjective beliefs about their symptoms significantly impact treatment satisfaction, prognosis, and adherence, it is essential to assess these perceptions. However, few studies have been conducted to assess patients’ subjective beliefs, their mental representation, of their disease. Therefore, this study aims to develop Interview that enable the utilization of patients’ cognitive representations of their mental illnesses in clinical practice.

**Objectives:**

The primary objective of this study is to develop a semi-structured interview and a self-report scale to evaluate patients’ mental representations of their illnesses. Subsequently, validate the reliability and validity of these tools as psychological assessments.

**Methods:**

An initial structure for both the semi-structured interview and self-report scale was established through a literature review of existing disease representation measurements. Subsequently, expert panel discussions and further literature reviews were conducted to refine the structure and content of both tools. Content validity for both the interview and self-report scale was assessed by a panel of nine experts and a group of ten students. Following this, the developed interview tool was subjected to a validity analysis with clinical patients using Missick’s six validity criteria(Content, Substantive, Structural, Generalizability, External, Consequential).

**Results:**

Content validity index (CVI) values for the overall structure indicated that all subdomains scored above 0.8, demonstrating the appropriateness of the interview tool’s five subdomains: symptoms, causes, temporal aspects, impact, and treatment and control. Content validity assessment for individual items revealed that some items within the “causes of the disease” subdomain, specifically stress-related factors, scored below 0.6, prompting necessary item modifications. All other factors achieved CVI scores of 0.6 or higher. Facial validity assessment yielded favorable results for all items in the self-report scale. All validity was demonstrated to be satisfactory.

**Conclusions:**

This study has provided evidence that the developed tools are reliable and valid instruments for measuring patients’ perceptions of their illnesses, offering a trustworthy means to assess these vital cognitive representations in clinical practice.

**Disclosure of Interest:**

None Declared

